# A case of fungal peritonitis in a patient with paramalignant ascites

**DOI:** 10.1016/j.mmcr.2024.100660

**Published:** 2024-07-14

**Authors:** Julia M. Berger, Felix Lötsch, Anna S. Berghoff, Wolfgang W. Lamm, Matthias Preusser, Georg Jeryczynski

**Affiliations:** a Division of Oncology, Department of Medicine I, Medical University of Vienna, Vienna, Austria; b Christian Doppler Laboratory for Personalized Immunotherapy, Department of Medicine I, Medical University of Vienna, Vienna, Austria; c Clinical Division of Laboratory Medicine, Medical University of Vienna, Vienna, Austria

**Keywords:** Fungal peritonitis, Neuroendocrine carcinoma, Ascites, *Candida albicans*, Caspofungin

## Abstract

Here, we present the case of a patient with a metastatic neuroendocrine tumor with cytologically negative ascites treated for spontaneous bacterial peritonitis (SBP). Ascitic cultures remained negative for bacterial growth but were positive for *Candida albicans* 8 days after SBP diagnosis. ß-D-glucan was only positive in ascites, while being negative in blood. Blood cultures remained negative throughout the whole admission. Fungal peritonitis presumably originated from an impending bowl perforation or an increasing vascular permeability caused by an increase in VEGF secondary to diffuse infiltration by the underlying malignant disease.

## 1 Introduction

Ascites is a common complication of many solid tumors affecting up to 21 percent of patients [1]. In contrast to ascites due to portal hypertension such as in liver disease, malignant ascites commonly presents as an exudate with high protein levels [2]. As the ascitic protein also contains opsonins, spontaneous bacterial peritonitis (SBP) is a rather uncommon phenomenon in these patients. Here, we present a case of a patient suffering from a neuroendocrine tumor and ascites, who developed fungal peritonitis (FP).

## 2 Case presentation

This is a case presentation of a male in his early fifties diagnosed with metastatic small bowel neuroendocrine tumor G2 seven months prior to hospital admission for the insertion of a permanent ascites drain. At time of admission, the patient had tumor lesions in the liver, lymph nodes and presented with peritoneal carcinomatosis. About one month after tumor diagnosis and 9 months prior to admission, treatment with somatuline was initiated and was still ongoing at time of hospital admission. Additionally, the patient underwent three peptide radio receptor therapies, the last one a month prior to hospital admission.

About one and a half months prior to hospital admission the patient presented with symptomatic ascites accumulation and underwent five paracenteses in one-to-two-week intervals. The ascites presented as chylic fluid, with characteristics of a transudate (serum-ascites-albumin-gradient, SAAG: 22.2) and with repeatedly negative cytology despite the presence of peritoneal carcinomatosis. Stenosis of the superior mesenteric vein by the peritoneal carcinomatosis was speculated to be the underlying cause for ascites formation. As 8.2–9.5 L of fluid were drained at each paracentesis, the decision was made to implant a permanent, tunneled catheter to allow the patient to independently manage ascites drainage according to symptom burden.

Upon admission for catheter insertion, the patient presented with fatigue and a reduced overall performance status. Accordingly, inflammation parameters were elevated (c-reactive protein CRP: 26.4 mg/dL, leukocytes: 14.06 × 10^9^/L). To rule out any infectious processes in the peritoneal cavity, a diagnostic paracentesis with ascites cultures was performed which revealed an ascitic neutrophil count (ANC) of 1.99 × 10^9^/L. Spontaneous bacterial peritonitis (SBP) was diagnosed and empirical antibiotic treatment with ceftriaxone was started immediately (day 0). Blood, urine and stool cultures failed to reveal an infectious focus.

On day 1 post SBP diagnosis a restaging computer tomography (CT) was performed and discussed at the interdisciplinary tumor board, showing the known tumorous lesions surrounding the mesenteric root to be stable in size according to RECIST criteria and no signs of abdominal perforation or other discernible causes for peritonitis [3]. However, in comparison with a PET-CT scan performed seven weeks prior, the superior mesenteric vein showed further signs of occlusion due to minimally progressive peritoneal carcinomatosis.

As the inflammation parameters did not decrease significantly and the patient developed fever on day 3 post SBP diagnosis, the antibiotic treatment was escalated to meropenem in combination with linezolid.

Under this treatment, the overall status of the patient improved, inflammatory parameters mildly decreased (CRP: from 26.4 mg/dL on day 1–21.55 mg/dL on day 6) and another diagnostic paracentesis on day 6 post SBP diagnosis showed decreasing neutrophile counts (ANC: 0.9 × 10^9^/L).

**Fig. 1:**
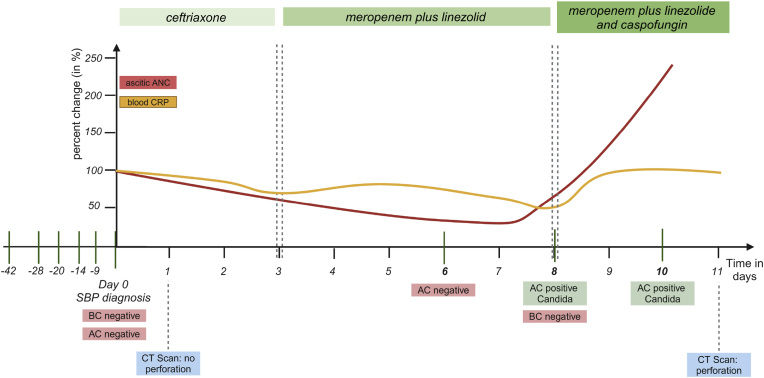
Clinical course of a patient with fungal peritonitis from the first to the last paracentesis (AC ascites culture, ANC ascitic neutrophil count, BC blood culture, CRP c-reactive protein, CT computer tomography, SBP spontaneous bacterial peritonitis).

On day 8 post SBP diagnosis, a follow up paracentesis was performed now revealing a renewed increase in ascitic neutrophils (ANC: 1.3 × 10^9^/L). Ascites cultures obtained during this paracentesis were positive for *Candida albicans* while the blood culture remained negative. The antibiotic therapy was immediately escalated with additional caspofungin (50 mg intravenously once daily). To rule out systemic candidiasis, ß-D-glucan was measured in blood on day nine, which remained negative. Another follow up paracentesis was performed on day 10 post SBP diagnosis, still presenting with a positive culture for *Candida albicans* (Fig. 1).

After an initial decrease on inflammatory parameters following initiation of antifungal therapy, and clinical improvement, the patient presented with a severely reduced performance status and distended abdomen on day 11 after SBP diagnosis. Abdominal CT was repeated and revealed mesenteric ischemia as well as small bowel perforation due to de novo occlusion of the superior mesenteric artery caused by the tumor bulk. Emergency surgical exploration was performed resulting in ileocecal resection. Two days later during a second look operation, another 40 cm of small bowel had to be resected to allow for the creation of an ileostomy. Despite these efforts, the patient's condition did not improve, and he developed septic multiorgan failure and passed away 18 days after initial SBP diagnosis.

## 3 Translational analysis

**Fig. 2:**
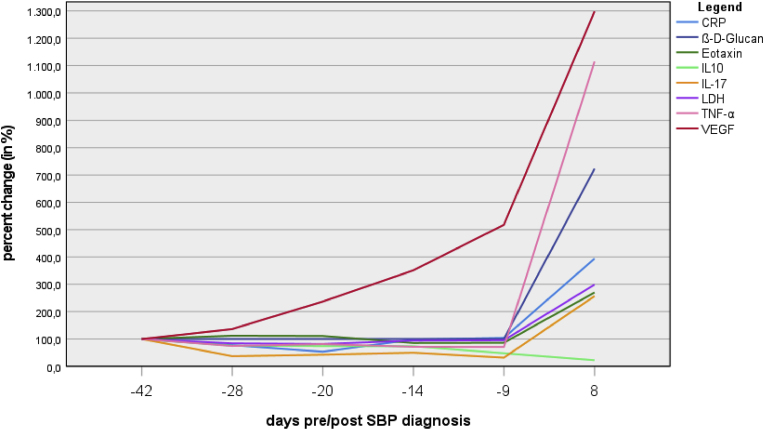
Change of cytokines pre and post SBP diagnosis in percent (CRP c-reactive protein, IL interleukin, LDH lactate dehydrogenase, SBP spontaneous bacterial peritonitis, TNF-α tumor necrosis factor-α, VEGF vascular endothelial growth factor).

As retrospective, experimental analyses, cytokine and ß-D-glucan measurements in ascites were performed in samples from several days before and one time after SBP diagnosis (days −42, −28, −20, −14, −9, 8). Proinflammatory cytokines could be measured in all available ascites samples, where a trend towards an increase in pro-inflammatory cytokines (eotaxin, IL-17, TNF-α, VEGF, CRP, LDH) over time with a visible spike in inflammation after SBP diagnosis was observed (Fig. 2). Concordantly, levels of the anti-inflammatory cytokine IL-10 dropped over the observed time period. ß-D-glucan could only be identified after SBP diagnosis on day 8 when the ascites culture was positive for *Candida albicans* (224.15 pg/mL). After SBP diagnosis, the leucocyte composition of the ascitic fluid changed from predominantly monocytes (45–75 %) to neutrophile dominance (60–89 %).

## 4 Discussion

Spontaneous bacterial peritonitis are infections of the peritoneal cavity without a surgically treatable focus, which are commonly treated by third-generation cephalosporins as empirical, first-line antibiotics. However, they fail to resolve the infection in 7–17 % of cases [4,5]. Even though their incidence is low, fungi may be causative pathogens in case of cephalosporin failure. Fungal peritonitis (FP) is a phenomenon mostly attributed to patients with ascites due to liver cirrhosis or patients undergoing peritoneal dialysis [6]. In patients with cirrhotic ascites, fungal infections are the underlying cause in around four percent of spontaneous peritonitis cases [6,7]. Reports mostly identified *Candida* *spp.* and *Cryptococcus neoformans* as pathogens [7].

The experience in this case presents in accordance withx these studies as the empirical treatment with ceftriaxone, initiated immediately after peritonitis diagnosis, failed to lower inflammation parameters and improve the patients’ clinical status sufficiently. After three days and a febrile episode of the patient, the antibiotic treatment was escalated to meropenem and linezolid which only slightly improved inflammation parameters. Only after eight days the ascites culture was positive and the antifungal treatment with caspofungin was initiated. This delay in causative treatment due to low clinical awareness for FP may explain the high mortality in patients with FP. One study reports a one-month-mortality rate as high as of 73.3 %, compared to a 28.7 % mortality in patients with bacterial peritonitis [7]. Also, pharmacokinetics needs to be considered: the penetration of caspofungin into the peritoneal cavity in critically ill patients is only 33 %. This indicates that in FP a dose escalation of 100mg as a loading dose may be necessary to ensure adequate, local caspofungin concentrations [8].

In fact, the patient only had a positive ascites culture of *Candida* on day 8 after peritonitis diagnosis. Indeed, the same species was identified in a previous stool culture which was deemed non-pathogenic and therefore, not treated as it is considered insignificant in clinical routine [9]. In general, spontaneous peritonitis is caused by translocation of gut microbiota through the blood stream [10]. As fungi are much larger in size than bacteria, fungal translocation across the gut mucosa requires a higher intestinal permeability which is rather common in late cirrhosis stages [11]. In our patient, the levels of the vascular endothelial growth factor (VEGF) increased over time. Indeed, VEGF was identified to be an important contributor to ascites formation in patients with malignant diseases [12]. Here, this increased vascular permeability may also facilitate the passage of germs through the intestinal lining into the interstitial space [10]. Measurement of ß-D-glucan in ascites, while not routine practice, deserves attention as a possible biomarker for FP and may be a possible target for future studies. To date, screening for ß-D-glucan is mostly performed in serum to test for invasive fungal infections. However, it is only positive in intraabdominal candidiasis in 10–20 % [13,14]. Intraabdominal ß-D-glucan was already shown to facilitate the exclusion of intraabdominal candidiasis and may be of use in other indications as well [15]. As fungal culture is the gold standard to test for peritoneal candidiasis, the measurement of ß-D-glucan may be helpful when suspecting fungal peritonitis until culture results are available. Still, additional studies have to be performed until this test can be used in clinical routine, in particular as there are currently no validated assays available that measure ß-D-glucan specifically in ascites.

As the patient presented with a bowl perforation on day 11 after SBP diagnosis, a contamination with feces may also be discussed even though perforation at the onset of peritonitis seems unlikely. Initially, bowl perforation was ruled out at day 1 after SBP diagnosis with the restaging CT and no fecal contamination was evident in the ascitic fluid at the paracentesis one day prior to diagnosis of perforation. Diagnostic work-up of this paracentesis however showed a sharp increase in ANC and LDH levels, possibly indicating an impending perforation. Chronic ischemia in the small bowel wall caused by chronic occlusion of the superior mesenteric vein and increasing stenosis of the artery by the tumorous bulk and minimal progression of the peritoneal carcinomatosis may have increased permeability and facilitated fungal translocation into ascitic fluid. However, it will have to remain speculation whether fungal transmucosal translocation was the initiating step of the SBP, or whether a possible occult perforation caused the ascitic candidiasis.

This is one of the few available reports of a fungal peritonitis as an extremely rare complication in ascites in a patient with an underlying malignancy [16]. In general, the ascites of this patient was more similar to a cirrhotic than a classically malignant one: cytology was always negative, SAAG and LDH gradient rather resembled a transudate even though there was a radiologic proof of peritoneal carcinomatosis. This highlights the heterogeneous causes of ascites in patients with solid tumors and the possibility of these patients to develop infectious peritonitis similar to ascites due to other causes.

## Conflict of interest

ASB has research support from Daiichi Sankyo (≤10000€), Roche (>10000€) and honoraria for lectures, consultation or advisory board participation from Roche Bristol-Meyers Squibb, Merck, Daiichi Sankyo (all <5000€) as well as travel support from Roche, Amgen and AbbVie.

MP has received honoraria for lectures, consultation or advisory board participation from the following for-profit companies: Bayer, Bristol-Myers Squibb, Novartis, Gerson Lehrman Group (GLG), CMC Contrast, GlaxoSmithKline, Mundipharma, Roche, BMJ Journals, MedMedia, Astra Zeneca, AbbVie, Lilly, Medahead, Daiichi Sankyo, Sanofi, Merck Sharp & Dome, Tocagen. ESB has honoraria for lectures, consultation or advisory board participation from Servier.

All other authors report not conflict of interest concerning this specific publication.

## Ethics approval and consent to participate

The patient was treated according to best clinical practice and current treatment guidelines throughout his whole clinical course of disease from diagnosis onwards at our tertiary care center. The translational research of this study was approved by the Ethics Committee of the Medical University of Vienna (vote number 2100/2022) and performed according to the Declaration of Helsinki and its Amendments. Consent to participate in this study was waived.

## Funding information

The financial support by the Austrian 10.13039/501100012416Federal Ministry for Digital and Economic Affairs, the 10.13039/100010132National Foundation for Research, Technology and Development and the 10.13039/501100006012Christian Doppler Research Association is gratefully acknowledged.

## Additional information

### Methods

Cytokine levels were be quantified using a multiplex immunoassay (Biorad, Hercules, California, USA) using the Luminex technology (Austin, Texas, USA). ß-D-glucan was measured with the Fungitell assay (Associates of Cape Cod, Inc. [ACCI], East Falmouth, MA, USA).

## Data availability

Data of this study is available from the corresponding author upon reasonable request.

## CRediT authorship contribution statement

**Julia M. Berger:** Writing – review & editing, Writing – original draft, Visualization, Validation, Software, Project administration, Methodology, Investigation, Formal analysis, Data curation, Conceptualization. **Felix Lötsch:** Writing – review & editing, Validation, Methodology, Formal analysis. **Anna S. Berghoff:** Writing – review & editing, Validation, Resources, Funding acquisition. **Wolfgang W. Lamm:** Writing – review & editing, Validation, Resources. **Matthias Preusser:** Writing – review & editing, Validation, Resources, Funding acquisition. **Georg Jeryczynski:** Writing – review & editing, Validation, Resources, Funding acquisition.
